# Single nucleotide polymorphism rs11614913 associated with CC genotype in miR-196a2 is overrepresented in laryngeal squamous cell carcinoma, but not salivary gland tumors in Polish population

**DOI:** 10.1007/s13353-018-0445-6

**Published:** 2018-04-29

**Authors:** Marcin Skalski, Adam Ustaszewski, Katarzyna Jaskiewicz, Katarzyna Kiwerska, Malgorzata Wierzbicka, Hanna Klimza, Reidar Grenman, Maciej Giefing

**Affiliations:** 1 Institute of Human Genetics, Polish Academy of Sciences, Poznan, Poland; 2 Department of Tumor Pathology, Greater Poland Cancer Center, Poznan, Poland; 3 Department of Otolaryngology and Laryngological Oncology, Poznan University of Medical Sciences, Poznan, Poland; 4 Department of Otorhinolaryngology – Head and Neck Surgery and Department of Medical Biochemistry, Turku University Hospital and University of Turku, Turku, Finland

**Keywords:** LSCC, miRNA-196-a2, Salivary gland tumor, rs11614913, Genotyping

## Abstract

The miRNA-196a2 has shown significance in the development of various neoplasms, including head and neck squamous cell carcinoma (HNSCC). The oncogenic functionality of this miRNA is mediated via its potential to target annexin A1 mRNA, a tumor suppressor gene involved in inhibition of the NF-κB pathway. Interestingly, recent data indicate a susceptibility for aforementioned neoplasms in patients with the CC genotype vs the CT and TT genotypes of the rs11614913 SNP located within the DNA sequence of the miR-196a2 that results in elevated expression of the gene. To further investigate this phenomenon, we genotyped this SNP in 40 patients with laryngeal squamous cell carcinoma (LSCC), the most common tumor of the head and neck region and 60 patients with salivary gland tumors (SGT) that show a yet unexplained incidence increase in the last two decades. In agreement with previous reports, we have identified a statistically significant (*p* < 0.05) overrepresentation of the CC genotype in LSCC patients and demonstrated in LSCC cell lines that it results in elevated expression of miR-196a2 as compared to cell lines with the TT genotype of the respective SNP. Importantly, none of these correlations was found in patients with SGT. These findings underline the importance of the SNP rs11614913 for LSCC development in the Polish population and moreover highlight the different genetic background of the two studied neoplasms of the head and neck region.

**Electronic supplementary material:**

The online version of this article (10.1007/s13353-018-0445-6) contains supplementary material, which is available to authorized users.

## Introduction

Tumors of the head and neck are highly prevalent worldwide. In this group, > 90% of tumors are represented by head and neck squamous cell carcinoma (HNSCC) of oral cavity, oropharynx and larynx, with known etiological factors including tobacco smoking, alcohol consumption, and human papilloma virus (HPV) infection (Vigneswaran and Williams 2014). Recently, growing incidence of the benign salivary gland tumors (SGT) is observed in the head and neck region that currently constitute 3–4% of head and neck tumors and are expected to rapidly grow in the next decades. Two most common SGTs—pleomorphic adenomas (PA) and Warthin tumors (WT)—constitute 87% parotid gland tumors (Wierzbicka et al. 2010). In contrast to HNSCC, the main causes of SGT are not well known, although factors like radiation, exposure to nickel, cement dust, asbestos, and nutrition have been suggested (Barnes et al. 2005). It is anticipated that also genetic factors contribute to SGT but currently this field remains significantly understudied.

In this study, we intended to analyze the frequency of the SNP rs11614913, known to affect the functioning of the oncogenic miR-196a2, in the two types of head and neck tumors, that is laryngeal squamous cell carcinoma (LSCC) and SGT. MiR-196a2 is overexpressed in many malignancies, including HNSCC, breast, lung, and pancreatic cancers (Christensen et al. 2010). The oncogenic functionality of this miRNA is mediated via its potential to target the annexin A1 mRNA, which is reported as a tumor suppressor. Annexin A1 negatively regulates NF-κB pathway, aberrantly expressed in many cancers (Zhang et al. 2010). Moreover, miR-196a2 targets *DFFA* (DNA fragmentation factor subunit alpha) responsible for apoptosis and downregulated in esophageal carcinoma and colon cancer (Fawzy et al. 2017). In relation to the role of miR-196a2, the SNP rs11614913 located in the premature sequence of the miRNA is reported to play a role in processing of the molecule, positively influences its expression and modulates the risk of tumor formation. Data from in vitro experiments with transfection of cell lines using vectors carrying the C or T variants, respectively, showed an increase in expression of this miRNA from the C variant vector (Hoffman et al. 2009).

## Results and discussion

**Fig. 1:**
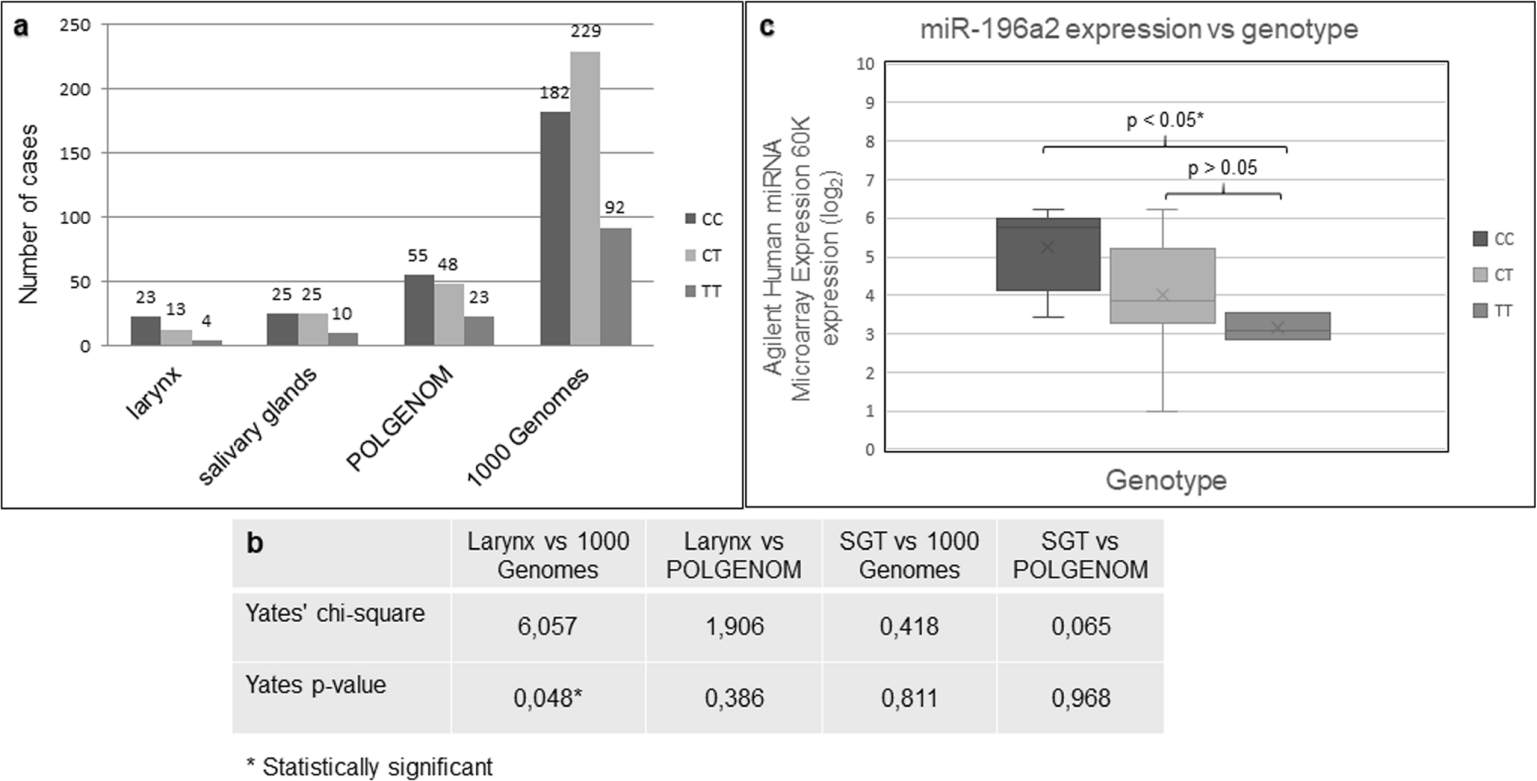
**a** Frequency of rs11614913 SNP genotypes in patients with LSCC (larynx), SGT (salivary glands) and in control groups—POLGENOM (Boguszewska-Chachulska et al. 2016) and 1000 Genomes (The 1000 Genomes Project Consortium 2015). DNA for genotyping was isolated from peripheral blood of patients with LSCC (*n* = 40) and SGT (*n* = 60) (*n* = 36 pleomorphic adenoma and *n* = 24 Warthin tumor). DNA was isolated using phenol-chloroform extraction and MagCore*®* Nucleic Acid Extraction Kit. Blood samples were collected in the Department of Otolaryngology of the University of Medical Sciences in Poznan, Poland, with the approval of the ethics institutional review board at the University of Medical Sciences in Poznan, Poland (No. 449/17). Informed consent was obtained from all subjects. Standard Sanger sequencing protocol was used for genotyping. **b** Chi-square test results. Calculations based upon an assumption, that observed genotypes of the rs11614913 SNP in LSCC and SGT are in Hardy-Weinberg equilibrium compared to 1000 Genomes and POLGENOM populations, respectively. Results obtained using on-line chi-square calculation tool (http://quantpsy.org). **c** Comparison of miR-196a2 expression (Agilent Human miRNA Microarray Expression 60K, Atlas Biolabs GmbH, Berlin, Germany) in the 16 LSCC cell lines depending on the genotype of the rs11614913 SNP, based on expression profiles reported by Janiszewska et al. 2015

To analyze the distribution of the rs11614913 genotypes in a homogenous cohort of Polish (Caucasian) LSCC patients (*n* = 40), we performed genotyping by Sanger sequencing in peripheral blood samples using primers flanking the rs11614913 SNP (forward: *ACCCAGCAACCCAAAGTCTA*, reverse: *GAGAGGACGGCATAAAGCAG*). This analysis resulted in the identification of *n* = 23 (57.5%) homozygotes CC, *n* = 13 (32.5%) heterozygotes CT, and *n* = 4 (10%) homozygotes TT (Fig. 1a). In line with previous reports, this distribution showed higher occurrence of the homozygote CC genotype in LSCC patients compared to 1000 Genomes (The 1000 Genomes Project Consortium 2015) but not POLGENOM (Boguszewska-Chachulska et al. 2016) control populations (chi-square test with Yates correction *p* < 0.05 and *p* > 0.05, respectively) (Fig. 1b). The lack of significant differences in the analyzed distributions between LSCC patients and the POLGENOM control population can be at least to some extent explained by the low number (*n* = 126) of genotyped individuals in the POLGENOM database. In regard to these findings, we raised the question if there is a correlation between homozygote CC genotype and higher expression of miR-196a2 that was previously reported for breast, gastric, and non-small cell lung cancers in Chinese and Caucasian populations (Slaby et al. 2012, Hoffman et al. 2009, Zhang et al. 2012).

In order to find further evidence that C variant of rs11614913 SNP in LSCC increases the expression of miR-196a2, we genotyped 16 LSCC cell lines and correlated the results with previously published miRNA expression profiles (Agilent Human miRNA Microarray Expression 60K, Atlas Biolabs GmbH, Berlin, Germany) (Janiszewska et al. 2015). The genotypes in the 16 studied cell lines were as follows: *n* = 6 (37.5%) CC homozygotes, *n* = 7 (43.75%) CT heterozygotes, and *n* = 3 (18.75%) TT homozygotes. The correlation between genotype and expression of the miR-196a2 was evaluated using the Wilcoxon test. By this analysis, we have confirmed that the CC genotype results in a statistically significant higher expression of the miR-196a2 compared to TT genotype (65% higher, *p* value < 0.05), but not compared to CT genotype (Fig. 1c). Encouraged by these findings, we next analyzed if a similar relationship can be observed in patients with SGT. Sanger sequencing based genotyping of peripheral blood samples from 60 patients with SGT resulted in identification of *n* = 25 (41.67%) CC homozygotes, *n* = 25 (41.67%) CT heterozygotes, and *n* = 10 (16.67%) TT homozygotes. In contrast to the observation made for LSCC, in this group, there were no statistically significant differences in the distribution of the SNP rs11614913 genotypes between these patients and the genotype frequencies reported in POLGENOM and 1000 Genomes databases (Fig. 1b).

Taking together, our results indicate a higher prevalence of the CC genotype (rs11614913) in LSCC patients compared to control population (1000 GENOMES), but lack of such association for SGT patients. Importantly, these data suggests that CC SNP rs11614913 genotype may be a potentially predisposing factor for development of LSCC, but not for SGT in Polish population. These intriguing findings suggest different genetic background of these diseases despite the exposition to the same carcinogens occurring in tobacco and alcohol, as well as similar tumor sites. This finding is an argument that different tumors of the head and neck region show significant genetic differences and should be regarded as separate entities. Moreover, such observations are in agreement with recently proposed WHO reclassifications in other neoplasms, for example, in lung cancers (Travis et al. 2015). It also suggests that future classification methods as well as targeted therapy should be based on genetic background of tumors rather than site or histologic subtype (Bunn et al. 2013).

## Electronic supplementary material

ESM 1 (XLSX 14 kb)
